# Trabectedin plus pegylated liposomal doxorubicin in recurrent ovarian carcinoma after poly-ADP-ribose polymerase inhibitors progression: A real-world single-center experience

**DOI:** 10.1016/j.gore.2026.102198

**Published:** 2026-09-11

**Authors:** Naroa Amondarain Amenabar, Karmele Mujika Eizmendi, Laura Gabirondo Amenabar, Marina Crespo Cruz, Iker Muñagorri Quiles, Elide Gutiérrez San José, Malena García Álvarez, Iera Arrondo Arizmendi, Nerea Bengoa Fernández, Javier Martínez Amorena, Maddi Sánchez Urrestarazu, Larraitz Egaña Otaño, Cristina Churruca Galaz

**Affiliations:** a Medical Oncology Department, Hospital Universitario Donostia, Donostia, Spain; b Medical Oncology Department, University College London Hospitals NHS Foundation Trust, London, UK

**Keywords:** Recurrent ovarian cancer, Trabectedin-pegylated liposomal doxorubicin, PARP inhibitors, Platinum sensitivity

## Abstract

**Background:**

Platinum-based chemotherapy remains the cornerstone of treatment for recurrent ovarian cancer; however, optimal treatment sequencing after progression on PARP inhibitors (PARPi) remains uncertain, particularly in patients with partially platinum-sensitive disease.

**Objective:**

to evaluate the efficacy, safety, and subsequent treatment patterns associated with trabectedin plus pegylated liposomal doxorubicin (PLD) in patients with recurrent ovarian cancer who had progressed after prior PARPi exposure.

**Methods:**

This retrospective, single-center real-world study included patients with recurrent ovarian cancer, a platinum-free interval of 6–12 months, and progression after prior PARPi exposure.

**Results:**

Twenty-five patients were included. The median age was 68.8 years, median platinum-free interval was 7.0 months, and patients had received a median of three prior lines of therapy. The objective response rate (ORR) was 24.0% and disease control rate (DCR) was 64.0%. Median progression-free survival (PFS) and overall survival (OS) were 7.9 and 19.9 months, respectively. Following progression on trabectedin plus PLD, 76.0% of patients received subsequent platinum-based chemotherapy, with an ORR of 33.3% among those receiving immediate platinum rechallenge. Toxicities were predominantly hematological, with no unexpected safety signals.

**Conclusions:**

In this real-world, heavily pretreated post-PARPi population with partially platinum-sensitive disease, trabectedin plus PLD demonstrated clinically meaningful activity with an acceptable safety profile. In a setting where available evidence remains limited and heterogeneous, these findings provide additional clinically relevant data in a well-defined subgroup and support its use as a feasible treatment option in selected patients. Prospective studies are warranted to further define optimal treatment sequencing after PARPi exposure.

## Highlights

• Trabectedin plus PLD shows activity after PARP inhibitor progression.
• Disease control reaches 64% in partially platinum-sensitive ovarian cancer.
• Median progression-free and overall survival reach 7.9 and 19.9 months.
• Platinum rechallenge after trabectedin plus PLD yields a 33.3% response rate.
• No toxicity-related discontinuations occur despite frequent neutropenia.

## 1 Introduction

Ovarian cancer is one of the most common gynecologic malignancies and remains the leading cause of death among gynecologic cancers in developed countries, with approximately 200,000 deaths reported worldwide in 2022 (Bray et al., 2024; Siegel et al., 2024). It is frequently diagnosed at an advanced stage (FIGO III–IV), largely due to the absence of effective screening strategies (González-Martín et al., 2023).

Complete cytoreductive surgery combined with platinum-based chemotherapy has long represented the standard of care for advanced ovarian cancer. Bevacizumab was introduced as maintenance therapy in 2011, and poly-ADP-ribose polymerase inhibitors (PARPi) were incorporated into recurrent disease management in 2014, particularly in tumors harboring *BRCA* mutations or showing homologous recombination deficiency (HRD) (Batalini et al., 2023; Boccia et al., 2024; Pignata et al., 2025). Subsequent pivotal studies such as SOLO-1, PRIMA, and PAOLA-1 demonstrated significant clinical benefit in the first line maintenance setting, especially in patients with *BRCA* mutations and/or HRD-positive tumors, establishing PARPi as standard maintenance therapy in the first-line treatment of advanced ovarian cancer (Caruso et al., 2023; Liu et al., 2024; Madariaga et al., 2019; Perez-Fidalgo et al., 2024; Redondo et al., 2023; Tew et al., 2020). Clinical practice guidelines developed by the European Society for Medical Oncology (ESMO) (González-Martín et al., 2023), the National Comprehensive Cancer Network (NCCN) (Liu et al., 2024) and the American Society of Clinical Oncology (ASCO) (Tew et al., 2020) recommend PARPi as maintenance therapy for patients with BRCA1/2-mutated or BRCA1/2-wild-type/HRD-positive tumors who have no evidence of disease or have achieved a complete or partial response after first-line platinum–paclitaxel chemotherapy.

Despite optimal initial treatment, approximately 85% of patients with advanced-stage disease will relapse within 10 years (Perez-Fidalgo et al., 2024). At recurrence, therapeutic decisions depend on multiple factors, including histological subtype, *BRCA* mutational status and prior exposure to anti-angiogenic agents. The treatment-free interval following last platinum therapy (platinum-free interval, TFIp) remains an important determinant of subsequent management, with platinum rechallenge generally being preferred in platinum sensitive disease (TFIp >6 months) when feasible (González-Martín et al., 2023; Liu et al., 2024; Madariaga et al., 2019; Perez-Fidalgo et al., 2024; Redondo et al., 2023). However, in the PARPi era, the traditional classification of platinum sensitivity based solely on the TFIp is being increasingly questioned, as responses to platinum may be influenced by prior PARPi exposure (González-Martín et al., 2023; Redondo et al., 2023; Vergote et al., 2022). Post hoc analyses of SOLO2 and retrospective series have reported reduced responses to platinum following PARPi treatment, suggestingpotential cross-resistance between platinum agents and PARPi (Baert et al., 2020; Cecere et al., 2020; Frenel et al., 2022). Proposed mechanisms underlying this cross-resistance include restoration of homologous recombination repair, *BRCA* reversion mutations, and stabilization of replication forks (McMullen et al., 2020).

Extending the TFIp through non‑platinum regimens has been proposed as a potential strategy to optimize subsequent treatment sequencing, although evidence supporting restoration of platinum sensitivity remains limited (Redondo et al., 2023; Vergote et al., 2022; Xu-Vuillard et al., 2024). In this context, the combination of trabectedin (Yondelis®) and pegylated liposomal doxorubicin (PLD) is the only non‑platinum doublet approved by the European Medicines Agency (EMA) for platinum-sensitive recurrent ovarian cancer; however, it is not approved by the U.S. Food and Drug Administration (FDA). Its approval in Europe is based on the phase III OVA-301 trial, in which trabectedin in combination with PLD showed improved progression-free survival compared with PLD alone, with the greatest benefit observed in patients with a TFIp of 6–12 months (Monk et al., 2012, 2010; Poveda et al., 2011). These findings are further supported by prospective and retrospective real-world studies evaluating trabectedin plus PLD in routine clinical practice (Monk et al., 2020; Pignata et al., 2021; Rubio et al., 2024; Runnebaum et al., 2018; Selle et al., 2020). Collectively, these studies have reported clinically meaningful antitumor activity and a manageable safety profile across heterogeneous patient populations, including heavily pretreated and older patients. However, as these studies were conducted largely before the widespread implementation of PARP inhibitor maintenance or included only a limited number of patients previously exposed to PARPi, they provide limited evidence regarding the effectiveness of trabectedin plus PLD in the contemporary post-PARPi setting.

The antitumor activity of trabectedin may be related to its unique mechanism of action. By binding to the minor groove of DNA and forming a bulky ternary complex with Nucleotide Excision Repair (NER) proteins, trabectedin interferes with transcription and induces double-strand DNA breaks, leading to apoptosis (Ray-Coquard, 2017). Unlike platinum compounds and PARPi, trabectedin does not rely on homologous recombination repair pathways, which may be relevant in tumors that have developed resistance to these agents. Preclinical data suggest that this mechanism could potentially extend the TFIp and facilitate subsequent platinum rechallenge (Monk et al., 2012; Poveda et al., 2011; Ray-Coquard, 2017).

However, this hypothesis has not been confirmed in prospective clinical trials. The INOVATYON trial, a large phase III trial that evaluated trabectedin plus PLD versus carboplatin–PLD in patients with recurrent ovarian cancer with partial platinum sensitivity (TFIp 6–12 months) did not achieve its primary objective of superiority in OS over platinum ((Colombo et al., 2023). Despite this, median overall survival (OS) was comparable between the two treatment arms. It should be noted that this trial was conducted prior to the introduction of PARPis.

In the current context of widespread use of PARPi as maintenance therapy and given that key randomized trials of trabectedin plus PLD were conducted in the pre-PARPi era, real-world data in post-PARPi populations are limited. This retrospective study aims to evaluate the clinical outcomes of trabectedin plus PLD in patients with recurrent ovarian cancer and a TFIp of 6–12 months who had progressed after prior platinum-based chemotherapy and PARPi exposure. Efficacy and safety outcomes were assessed, together with an evaluation of subsequent systemic therapy following progression on trabectedin plus PLD, with particular focus on the use of platinum-based regimens administered after progression.

## 2 Materials and methods

### 2.1 Study design and data collection

This retrospective, observational, single-center case series was conducted at the Medical Oncology Department of the Clinical Management Unit of Gipuzkoa and included consecutive patients with recurrent ovarian cancer treated with trabectedin plus PLD after prior platinum-based chemotherapy and PARPi between October 2019 and December 2024.

Eligible patients were women aged ≥18 with histologically confirmed ovarian carcinoma and partially platinum-sensitive recurrence, defined as TFIp of 6–12 months, and without contraindications to treatment according to routine clinical practice. All patients had received PARP inhibitor maintenance therapy following at least one prior platinum-based chemotherapy regimen and had subsequently experienced disease progression before receiving trabectedin plus PLD. No patients were excluded beyond the predefined eligibility criteria.

TFIp was defined as the time from the last platinum-based chemotherapy dose to radiologically confirmed disease progression according to RECIST version 1.1. TFIp was calculated independently of PARPi exposure and was not redefined according to PARPi discontinuation.

PLD was administered at 30 mg/m^2^ as a 90-min intravenous infusion, followed by trabectedin at 1.1 mg/m^2^ as a 3-h intravenous infusion, every 3 weeks. Premedication included 20 mg of intravenous dexamethasone administered 30 min before PLD infusion for antiemetic and hepatoprotective purposes. Treatment continued until radiological disease progression according to RECIST version 1.1 criteria or unacceptable toxicity. Dose modifications and delays followed the local marketing authorization and the treating clinician's best clinical judgment.

Tumor assessments were performed at baseline and subsequently every three treatment cycles or according to the physician's indication. Response evaluation was based on RECIST version 1.1 criteria and was performed by the treating physicians using routine clinical imaging.

Platinum rechallenge after progression on trabectedin plus PLD was decided by the treating physician based on clinical judgment, prior platinum response, performance status, and patient preference.

Follow-up began on the date of first trabectedin plus PLD administration and continued until the last assessment or death.

This study was conducted in accordance with Spanish regulations on observational studies, following the provisions of Royal Decree 957/2020. The study protocol was reviewed and approved by the Ethics Committee for Research with Medicinal Products of the Basque Country (CEIm-Euskadi, EOM2025011). As this was a retrospective study using anonymized data, informed consent was not required.

### 2.2 Data collection

Clinical and treatment data were extracted from electronic medical records and recorded in an anonymized database. All relevant variables were available in the records, with no additional data retrieval required. Only date of birth was retained as an identifying variable, while all other personal identifiers were excluded.

Collected variables included date of birth, diagnosis date, tumor histology, germline *BRCA* status, somatic *BRCA* status, HRD status, prior bevacizumab, prior PARPi use and duration, and interval from the last platinum-based treatment to trabectedin plus PLD. PARP inhibitor maintenance therapy was not counted as a separate line of therapy but was considered part of the preceding platinum-based treatment line.

Treatment variables included number of prior lines before trabectedin plus PLD, number of trabectedin plus PLD cycles, treatment start and end dates, response to trabectedin plus PLD according to RECIST version 1.1 criteria, and the date of progression during trabectedin plus PLD therapy. Toxicity was assessed according to the National Cancer Institute Common Terminology Criteria for Adverse Events (CTCAE), version 5.0, together with treatment delays and dose reductions. Follow-up data included subsequent treatment lines, the platinum regimen and cycles in patients receiving platinum-based therapy after progression on trabectedin plus PLD, response to platinum-based therapy according to RECIST version 1.1, dates of best response and progression on platinum-based treatment, last follow-up date and clinical status at last follow-up.

### 2.3 Endpoints

Primary endpoints assessed the antitumor activity of trabectedin plus PLD, including objective response rate (ORR, defined as the proportion of patients achieving complete or partial response according to RECIST version 1.1 criteria), disease control rate (DCR, defined as the proportion with complete response, partial response, or stable disease according to RECIST version 1.1 criteria), progression-free survival (PFS; time from first trabectedin plus PLD administration to documented disease progression or death), and overall survival (OS; time from first trabectedin plus PLD administration to death from any cause). Patients who had not experienced progression or death at final analysis, or who were lost to follow-up, were censored at last contact or last date known to be alive.

Secondary endpoints included subsequent systemic therapies after trabectedin plus PLD, number of subsequent treatment lines and proportion of patients receiving platinum-based chemotherapy. For patients receiving platinum as the immediate subsequent line, the platinum regimen administered and treatment outcomes (ORR and DCR) were further assessed.

Safety of trabectedin plus PLD was evaluated through adverse events graded according to the CTCAE, version 5.0, as well as dose reductions, treatment delays, and treatment discontinuations.

### 2.4 Statistical analysis

Continuous variables were described using mean, standard deviation, median, and range, when appropriate. Categorical variables were presented as frequencies and percentages. ORR were described using descriptive statistics, with corresponding 95% exact binomial confidence intervals. Time-to-event endpoints, including duration of response (DoR), PFS, and OS, were analyzed using the Kaplan–Meier method. All statistical analyses were conducted using SAS software (version 9.4).

## 3 Results

A total of 25 patients with recurrent ovarian cancer met the eligibility criteria and were included in the analysis. The median age was 68.8 years (range 53–84). Regarding *BRCA* status, 7 patients (28.0%) harbored a pathogenic germline or somatic *BRCA* mutation, while 18 patients (72.0%) were *BRCA* wild type. Most patients (80.0%) had previously received bevacizumab.

All patients had been previously exposed to PARPi: niraparib was the most frequently used agent (76.0%), followed by olaparib (24.0%). PARPi had been administered as maintenance therapy in first line treatment in 3 patients (12.0%), in second line in 14 patients (56.0%), and beyond the second line in 8 patients (32.0%). The median PARPi treatment duration was 8.0 months (range 3.0–27.0).

Before initiation of trabectedin plus PLD, patients had received a median of 3 prior systemic therapy lines (range 1–5). Regarding previous treatment regimens, 24 patients had received carboplatin plus paclitaxel (24/25, 96.0%), 22 patients (88.0%) carboplatin plus PLD and 5 patients (20.0%) carbopatin plus gemcitabine. One patient (4.0%) had been treated with paclitaxel and subsequently carboplatin in later treatment lines.

In addition, 3 patients (12.0%) had received immunotherapy/placebo in combination with chemotherapy as part of a clinical trial, including carboplatin plus paclitaxel in 1 patient and carboplatin plus PLD in 2 patients. One patient (4.0%) had been treated with atezolizumab plus an anti-CD25 antibody in the context of a clinical trial.

The median interval from initial diagnosis to first trabectedin plus PLD administration was 3.9 years (range 1.0–16.7). The median TFIp was 7.0 months (range 6–11).

Baseline characteristics of the participants are summarized in Table 1**.**

**Table 1:** Baseline characteristics.

Characteristic	Participants (*N* = 25)
**Age (years)**	
Mean ± SD	66.8 ± 8.8
Median (range)	68.8 (53–84)

***BRCA* Status**, n (%)	
Mutated	7 (28.0)
Wild type	18 (72.0)
**Prior bevacizumab**, n (%)	20 (80.0)

**Prior PARPi**, n (%)	
Niraparib	19 (76.0)
Olaparib	6 (24.0)

**PARPi treatment line**, n (%)	
1st line	3 (12.0)
2nd line	14 (56.0)
>2nd line	8 (32.0)
**PARPi treatment duration (months),** median (range)	8.0 (3.0–27.0)

**Time from diagnosis to first infusion of TRB + PLD**, years	
Mean ± SD	5.7 ± 3.9
Median (range)	3.9 (1.0–16.7)
**No. of previous lines before TRB + PLD**	
Mean ± SD	3 ± 1.0
Median (range)	3 (1–5)

Previous treatment regimens	
Carboplatin + paclitaxel	24 (96%)
Carboplatin + PLD	22 (88%)
Carboplatin + gemcitabine	5 (20%)
Paclitaxel	1 (4%)
Carboplatin	1 (4%)
Carboplatin + paclitaxel + immunotherapy/ placebo (clinical trial treatment)	1 (4%)
Carboplatin + PLD + immunotherapy/ placebo (clinical trial treatment)	2 (8%)
Atezolizumab + anti-CD25 antibody (clinical trial treatment)	1 (4%)

**TFIp (months)**	
Mean ± SD	7.6 ± 1.8
Median (range)	7.0 (6–11)

*BRCA*: breast cancer gene; PARPi: poly (ADP-ribose) polymerase inhibitor; TRB + PLD: trabectedin plus pegylated liposomal doxorubicin; TFIp: platinum-free interval; SD: standard deviation.

Patients received a median of 7 cycles of trabectedin plus PLD (range 1–27). The ORR was 24.0% (6 of 25 patients) and the DCR reached 64.0% (16 of 25 patients). All objective responses were partial responses. 10 patients (40.0%) had stable disease while progressive disease was observed as the best response in 7 patients (28.0%). Response status was not evaluable in 2 patients (8.0%, Table 2).

**Table 2:** Treatment history and response to TRB + PLD after PARPi exposure.

Characteristic	Participants (N = 25)
**TRB + PLD cycles administered**	
Mean ± SD	7 ± 6.0
Median (range)	7 (1–27)

**Overall response after TRB + PLD**, n (%)	
Partial Response	6 (24.0)
Stable Disease	10 (40.0)
Progressive Disease	7 (28.0)
NA/unknown	2 (8.0)
**ORR**, % (95% CI)	24 (9.4–45.1)
**DCR**, % (95% CI)	64.0 (42.5–82)

TRB + PLD: trabectedin plus pegylated liposomal doxorubicin; SD, standard deviation; NA, not available; CI, confidence interval; Objective response rate (ORR) was defined as the percentage of patients achieving either a complete or partial response. Disease Control Rate (DCR) corresponded to the percentage of patients with complete response, partial response or stable disease. ORR was calculated as (complete response + partial response) divided by the total number of ITT patients, whereas DCR was calculated as (complete response + partial response + stable disease) divided by the total number of ITT patients.

**Fig. 1:**
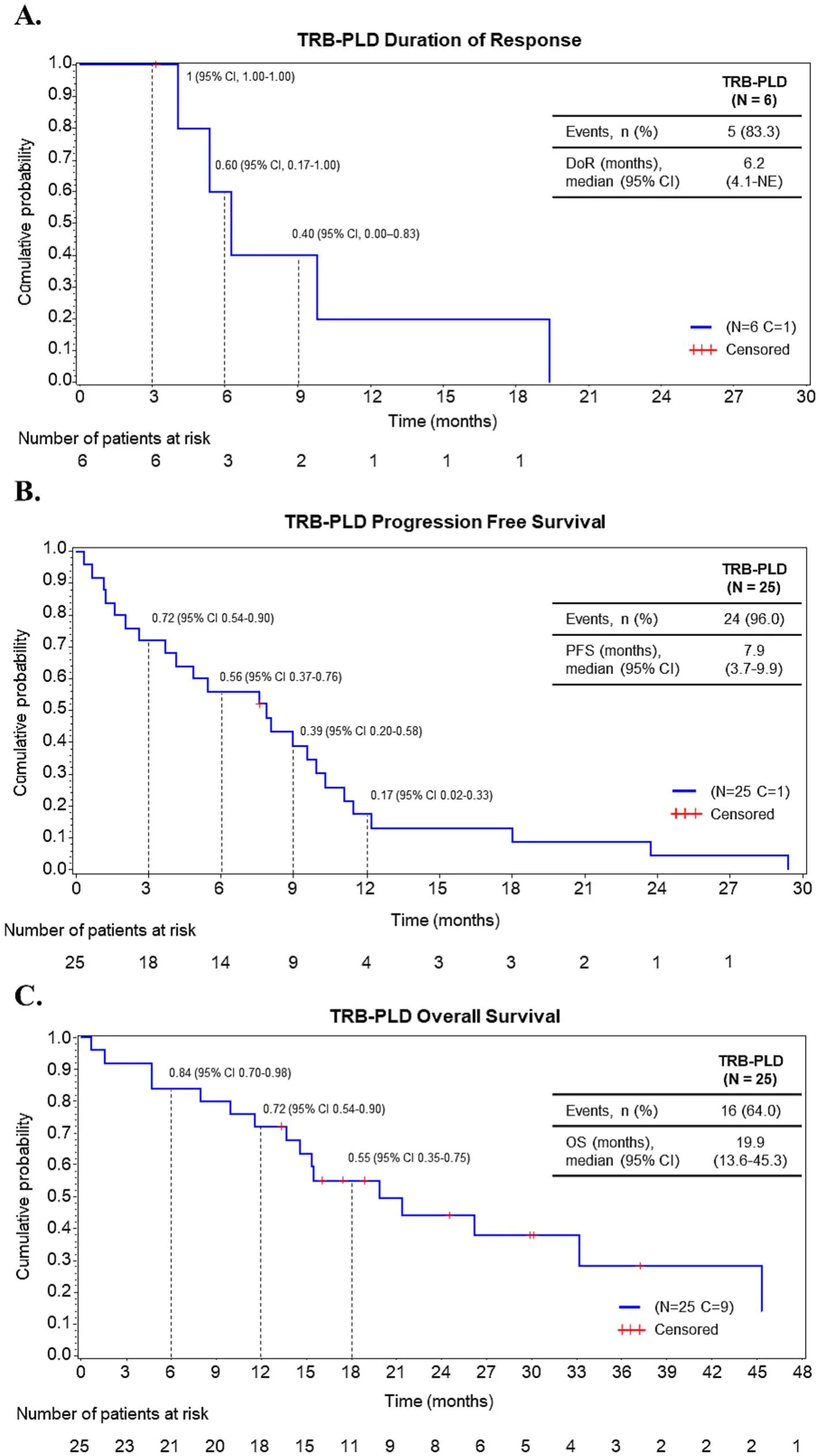
Kaplan–Meier curves for duration of response (A), progression-free survival (B), and overall survival (C) among patients receiving trabectedin plus pegylated liposomal doxorubicin following prior PARP inhibitor exposure. CI, confidence interval; DoR, duration of response; NE, not estimable; OS, overall survival; PFS, progression-free survival; TRB + PLD, trabectedin plus pegylated liposomal doxorubicin.

The median DoR was 6.2 months (95% CI, 4.1–NE). At 3 months, all responders remained progression-free, and at 9 months, 40.0% (95% CI, 0–82.9%) were still responding (Fig. 1A).

With a median follow up of 24.5 months, median PFS for the overall cohort was 7.9 months (95% CI, 3.7–9.9). At 12 months, 17.3% of patients remained progression-free (Fig. 1B). One patient was censored before documented disease progression and was still under follow-up at the time of data cut-off.

Median OS was 19.9 months (95% CI, 13.6–45.3). At 18 months, the estimated OS rate was 55.1%, (Fig. 1C). Nine patients (36.0%) were censored in the OS analysis, with 1 patient remaining alive at the end of follow-up.

After progression on trabectedin plus PLD, 19 patients (76.0%) received at least 1 subsequent line of systemic therapy. Six patients (24.0%) received 1 subsequent line, 8 patients (36.0%) received 2 lines and 7 patients (28.0%) received more than 2 additional lines of therapy.

After progression on trabectedin plus PLD, 19 patients (76.0%) received platinum-based chemotherapy at some point during subsequent treatment, and 15 patients (60.0%) received platinum as the immediate next line. Among these 15 patients, 2 (13.3%) received platinum monotherapy, 9 (60.0%) received carboplatin–gemcitabine, and 4 (26.7%) received carboplatin–PLD. The median number of cycles administered was 5.5. Objective responses were observed in 5 cases (33.3%), including one complete and four partial responses. Stable diseases were observed in two patients, while four patients experienced progressive disease. Response was not evaluable in 4 patients (Table 3).

**Table 3:** Subsequent therapy and response to immediate platinum after trabectedin plus pegylated liposomal doxorubicin.

Characteristic	Participants (N = 25)
**Further therapy No. of lines,** n (%)	
0	4 (16.0)
1	6 (24.0)
2	8 (32.0)
>2	7 (28.0)

**Further therapy with Platinum any line**, n (%)	
No	4 (16)
Unknown	2 (8.0)
Yes	19 (76.0)
Further Immediate Platinum (at progression)	15 (60.0)

**Further Immediate Platinum (*N* = 15)**	
**Platinum regimen,** n (%)	
platinum monotherapy	2 (13.3)
Carboplatin-gemcitabine	9 (60)
Carboplatin-PLD	4 (26.7)
**Platinum cycles administered**	
Mean ± SD	4.38 ± 2.06
Median (range)	5.5 (1.0–7.0)

**Overall Response Further Immediate Platinum**, n (%)	
Complete Response	1 (6.7)
Partial Response	4 (26.7)
Stable Disease	2 (13.3)
Progressive Disease	4 (26.7)
NA/unknown	4 (26.7)
**Objective Response Rate**, % (95% CI)	33.3 (11.8–61.6)
**Disease Control Rate**, % (95% CI)	46.7 (21.3–73.4)

CI, confidence interval; NA, not available.

Treatment-related adverse events are summarized in Table 4. Adverse events were common and mainly hematological. Grade 3–4 toxicities were predominantly neutropenia, which represented the most frequent severe adverse event. However febrile neutropenia was uncommon, it was observed only in one patient. Other grade 3–4 hematological toxicities, including anemia and thrombocytopenia, were infrequent.

**Table 4:** Safety profile of trabectedin plus PLD treatment.

	**Participants (*N* = 25)**
**Patients with trabectedin plus PLD reductions**, n (%)	
Yes	14 (56.0)
No	11 (44.0)
**No. of trabectedin plus PLD reductions**	
Mean ± SD	1.0 ± 1.0
Median (range)	1.0 (0.0–2.0)
Patients with trabectedin plus PLD delays, n (%)	
Yes	19 (56.0)
No	6 (24.0)
No. of trabectedin plus PLD delays, n (%)	
Mean ± SD	3.6 ± 3.9
Median (range)	4.0 (0–19)
**Adverse events associated with trabectedin plus PLD, CTCAE v5.0**, n (%)	
**Neutropenia**	21 (84.0)
1	1 (4.0)
2	1 (4.0)
3	10 (40.0)
4	9 (36.0)
**Anemia**	18 (72.0)
1	7 (28.0)
2	9 (36.0)
3	2 (8.0)
**Asthenia**	17 (68.0)
1	6 (24.0)
2	6 (24.0)
3	5 (20.0)
**Thrombocytopenia**	7 (28.0)
2	4 (16.0)
3	2 (8.0)
4	1 (4.0)
**Elevation of AST**	6 (24.0)
1	4 (16.0)
2	2 (8.0)
**Elevation of ALT**	7 (28.0)
1	6 (24.0)
2	1 (4.0)
**Nausea**	10 (40.0)
1	7 (28.0)
2	1 (4.0)
3	2 (8.0)
**Mucositis** 1 2 **Vomiting** 1 2 3	3 (12.0) 2 (8.0) 1 (4.0) 10 (40.0) 3 (12.0) 4 (16.0) 3 (12.0)

CTCAE: Common Terminology Criteria for Adverse Events; SD: Standard deviation.

Non-hematological adverse events were mostly of low grade. Asthenia and gastrointestinal toxicity were frequently reported but were generally mild to moderate in severity. Elevations in liver enzymes were common; however, these events were predominantly grade 1–2, transient, and rarely associated with clinically significant consequences or treatment discontinuation.

Six patients required hospitalization during treatment with trabectedin plus PLD. The reasons for hospitalization were vomiting (*n* = 2; one grade 3 and one grade 2), respiratory infection (*n* = 1; grade 2), febrile neutropenia with concomitant sepsis (*n* = 1; both grade 3), intestinal perforation (*n* = 1; grade 2), and intestinal subocclusion (*n* = 1; grade 3).

One patient died due to a secondary hematological malignancy attributed to prior chemotherapy exposure. This event was considered a serious adverse event but was not deemed related to trabectedin plus pegylated liposomal doxorubicin.

Dose modifications were common, mainly due to neutropenia. Dose reductions were required in 14 patients (56.0%), mainly because of neutropenia, whereas 19 patients (76.0%) experienced at least one dose delay. The median number of dose reductions was one (range, 0–2), and the median number of dose delays was four (range, 0–19). No treatment discontinuations due to toxicity occurred in this cohort.

## 4 Discussion

### 4.1 Summary of main results

This single-center retrospective study, provides descriptive real-world evidence on trabectedin plus PLD in patients with recurrent ovarian cancer progressing after platinum-based chemotherapy and PARPi therapy with a TFIp of 6–12 months. The study population was heavily pretreated, with a median of three prior lines of systemic therapy and high prior bevacizumab exposure.

*BRCA* status was determined in all participants: 28.0% carried a pathogenic germline or somatic mutation, while the remaining cases were *BRCA* wild type. Most patients (22/25) received PARPi in the second line or beyond, consistent with treatment patterns before the widespread adoption of first-line maintenance strategies.

Trabectedin plus PLD resulted in an ORR of 24.0% and a DCR of 64.0%, with median PFS and OS of 7.9 and 19.9 months, respectively. Following progression, 76.0% of patients received additional systemic therapy, and platinum-based chemotherapy was reintroduced in 19 patients. Among patients receiving immediate platinum rechallenge after trabectedin plus PLD, the ORR was 33.3%. The safety profile was consistent with known toxicities of the regimen, with hematologic adverse events, particularly neutropenia, being the most frequent grade 3–4 toxicity.

### 4.2 Results in the context of published literature

In the OVA-301 trial, trabectedin plus PLD demonstrated superior PFS compared with PLD monotherapy in patients with platinum-sensitive recurrent ovarian cancer (TFIp >6 months). A greater benefit was observed in the subgroup of patients with a TFIp of 6–12 months, although this was based on subgroup analysis. Importantly, most patients enrolled in OVA-301 received the study treatment in the second-line setting, whereas the population included in our study was more heavily pretreated (Colombo et al., 2023); Rubio et al., 2024). Despite greater pre-treatment, high prior bevacizumab exposure (80.0%) and prior PARPi exposure (100%), the ORR of 24.0% and the median PFS of 7.9 months observed in our cohort are broadly consistent with the outcomes reported in the partially platinum-sensitive subgroup of the OVA-301 (ORR 27% and PFS 7.4 months) (Poveda et al., 2011).

In the OVC-3006 study, trabectedin plus PLD demonstrated a higher ORR (46%) than PLD alone when used as third-line therapy for recurrent ovarian cancer, although this did not translate into a statistically significant improvement in OS. In that study, the median OS among patients with partially platinum-sensitive disease was 24.8 months (Monk et al., 2020). In our cohort, median OS of 19.9 months was observed, slightly lower than that reported in both OVA-301 (Monk et al., 2012) and OVC-3006 (Monk et al., 2020), which may reflect the more heavily pretreated population, higher median age and universal prior exposure to PARPi.

Importantly, both OVA 301 and OVC 3006 were conducted before widespread PARPi use, limiting direct comparability with our cohort.

As noted above, the INOVATYON trial did not achieve its primary objective of superiority in OS over platinum-based therapy in patients with partially platinum-sensitive recurrent ovarian cancer; nevertheless, median OS was comparable between the two treatment arms (Colombo et al., 2023)). INOVATYON addressed a different clinical question from that examined in the present study. Rather than evaluating the role of trabectedin plus PLD in the post-PARPi setting, it compared a non‑platinum strategy with platinum-based chemotherapy before PARP inhibitor maintenance became standard of care. Consequently, patients previously exposed to PARPi were not represented.

Real-world evidence in patients previously exposed to PARPi remains limited and heterogeneous. Vertechy et al. evaluated, in a case-control study, the efficacy of trabectedin plus PLD versus platinum-based therapy in platinum-sensitive ovarian cancer patients progressing during PARPi maintenance. They reported comparable clinical outcomes between strategies, although the platinum-treated cohort had a longer TFIp (>12 months), whereas the trabectedin plus PLD group had a TFIp of 6–12 months (Vertechy et al., 2023). More recently, in the MITO39 study, a multicenter case–control analysis, patients treated with trabectedin plus PLD after PARPi exposure were reported to have shorter PFS compared with PARPi-naïve patients. The non-randomized design of both studies limits interpretation. Importantly, while MITO39 provides comparative insights into the impact of prior PARPi exposure on trabectedin plus PLD outcomes, it does not specifically address treatment sequencing strategies.

In this context, our findings provide additional real-world data in a clinically well-defined subgroup of patients with partially platinum-sensitive disease (TFIp 6–12 months). Despite prior PARPi exposure and multiple previous lines of therapy, clinically meaningful activity and disease control were observed. Overall, these findings highlight the variability of outcomes in the post-PARPi setting and suggest that treatment efficacy may depend on clinical context and patient selection. While prior PARPi exposure may be associated with less favorable outcomes in some series, clinically relevant activity can still be observed in selected patients. However, all available data, including the present study, are derived from retrospective analyses, and more robust prospective studies are needed to better define the role of trabectedin plus PLD in this setting.

With regard to subsequent treatments, post-hoc analyses of OVA-301 conducted by Kaye et al. (2011) and Colombo et al. (2014) suggested that trabectedin plus PLD could delay the initiation of subsequent chemotherapy and extend the TFIp, potentially allowing later platinum rechallenge. In our cohort, more than half of patients received platinum rechallenge as the immediate subsequent line after trabectedin plus PLD, with an ORR of 33.3%. This rate is within or slightly above the range previously reported for partially platinum-sensitive patients treated with platinum rechallenge (27–33%) (Colombo and Gore, 2007). However, this observation is based on a small and heterogeneous subgroup and should be interpreted cautiously.

The NIMES-ROC study, a large European multicenter prospective study, evaluated the administration of trabectedin plus PLD in later treatment lines. In this study, patients received therapy beyond the second line and included elderly and heavily pretreated individuals. Trabectedin plus PLD retained activity and acceptable tolerability in those populations (Pignata et al., 2021), including a small subgroup previously exposed to PARPi (Pignata et al., 2022). Together with the present analysis, these findings suggest that this regimen may represent a feasible option in complex clinical scenarios that are often underrepresented in randomized trials.

Finally, the manageable safety profile of the treatment, with no unexpected adverse events reported, is also consistent with the results observed in the OVA-301 study (Monk et al., 2012) and other real-world experiences. Hematological toxicity, particularly neutropenia, was the most frequent grade 3–4 adverse event and was generally manageable with dose delays or reductions. Importantly, serious adverse events were infrequent and heterogeneous, and no treatment-related deaths were observed, supporting the feasibility of this regimen in an older and heavily pretreated population. Similar rates of dose modifications were reported in other studies such as NIMES-ROC or OVA-YOND (Pignata et al., 2021; Runnebaum et al., 2018).

### 4.3 Strengths and limitations

While real-world data on trabectedin plus PLD after PARPi exposure remain limited, the present study focuses on a strictly defined partially platinum-sensitive population (TFIp 6–12 months) uniformly exposed to PARPi. Reflecting routine clinical practice in a heavily pretreated population, this analysis provides a transparent and descriptive characterization of treatment outcomes and safety in this specific clinical setting.

However, several limitations must be acknowledged. The retrospective single-center design and the relatively small sample size (n = 25) limit the statistical power of the study and preclude any robust comparative conclusions.

Further limitation relates to patient selection and the lack of information on the denominator population of patients progressing after PARP inhibitor therapy during the study period. The database was designed to capture only patients treated with trabectedin plus PLD and did not systematically record patients receiving alternative treatments. Therefore, the total number of eligible patients and the proportion treated with other regimens cannot be determined.

The absence of a control group makes it difficult to determine whether the observed outcomes are attributable to trabectedin plus PLD or to patient selection and treatment sequencing. In addition, the cohort was heterogeneous with respect to prior lines of therapy, timing and duration of PARPi exposure, *BRCA* status, and subsequent treatments, all of which may influence outcomes. Information on homologous recombination deficiency (HRD) status was not available for most patients, as HRD testing was not routinely implemented in our healthcare system during the period in which these patients were treated. Subgroup analyses were not performed due to the small number of patients per subgroup and the substantial heterogeneity of clinical characteristics, which would have limited interpretability and robustness of the results.

OS estimates are limited by a relatively high rate of censoring. In contrast, censoring was minimal in the PFS analysis, as most patients experienced disease progression during follow-up. However, the retrospective assessment of progression and the non-uniform timing of imaging evaluations may still have affected the precision of progression timing and, consequently, the estimation of PFS.

Finally, although responses to platinum rechallenge were observed in a subset of patients, these findings are based on a small and heterogeneous group and should be considered exploratory.

### 4.4 Current implications and future research

In the current therapeutic landscape, where optimal sequencing after PARPi failure remains uncertain and available evidence remains limited and heterogeneous, non‑platinum strategies may retain a role in selected patients with partially platinum-sensitive recurrent ovarian cancer. The present findings provide descriptive real-world evidence supporting the feasibility of trabectedin plus PLD as a non‑platinum option within the therapeutic sequence. These findings should be interpreted in the context of the study population, which was restricted to patients with a platinum-free interval of 6–12 months and consisted almost exclusively of patients with high-grade serous ovarian carcinoma. Therefore, the results should not be extrapolated to patients with longer platinum-free intervals or other histological subtypes.

In this cohort, responses to subsequent platinum-based therapy were observed in a subset of patients, consistent with previously reported post-hoc and real-world observations. However, these findings should be interpreted cautiously and considered exploratory, as no causal inference regarding restoration of platinum sensitivity can be drawn.

Future research should focus on prospective, multicenter studies specifically designed to address treatment sequencing after PARPi exposure, including the role of non‑platinum regimens in patients with intermediate TFIps. Integration of biomarker-driven approaches, improved characterization of resistance mechanisms, and incorporation of translational endpoints may help identify patient subgroups most likely to benefit from these strategies.

## 5 Conclusions

This single-center real-world analysis provides descriptive real-world evidence regarding the use of trabectedin combined with PLD in heavily pretreated patients with recurrent ovarian cancer previously exposed to PARPi and with a platinum-free interval of 6–12 months. The regimen showed clinical activity and a manageable safety profile in this challenging population.

In a setting where available evidence remains limited and heterogeneous, these findings provide additional clinically relevant data in a well-defined subgroup of patients.

While responses to subsequent platinum rechallenge were observed in a subset of patients, given the exploratory nature of these findings, they should be interpreted with caution. Prospective multicenter studies are needed to further validate these results and to better define the optimal sequencing of non‑platinum regimens and platinum-based therapies in the post PARPi setting.

## CRediT authorship contribution statement

**Naroa Amondarain Amenabar:** Writing – review & editing, Resources, Data curation, Conceptualization. **Karmele Mujika Eizmendi:** Writing – review & editing, Resources, Data curation, Conceptualization. **Laura Gabirondo Amenabar:** Writing – review & editing, Resources, Data curation, Conceptualization. **Marina Crespo Cruz:** Writing – review & editing, Resources, Data curation, Conceptualization. **Iker Muñagorri Quiles:** Writing – review & editing, Resources, Data curation, Conceptualization. **Elide Gutiérrez San José:** Writing – review & editing, Resources, Data curation, Conceptualization. **Malena García Álvarez:** Writing – review & editing, Resources, Data curation, Conceptualization. **Iera Arrondo Arizmendi:** Writing – review & editing, Resources, Data curation, Conceptualization. **Nerea Bengoa Fernández:** Writing – review & editing, Resources, Data curation, Conceptualization. **Javier Martínez Amorena:** Writing – review & editing, Resources, Data curation, Conceptualization. **Maddi Sánchez Urrestarazu:** Writing – review & editing, Resources, Data curation, Conceptualization. **Larraitz Egaña Otaño:** Writing – review & editing, Resources, Data curation, Conceptualization. **Cristina Churruca Galaz:** Writing – review & editing, Resources, Data curation, Conceptualization.

## Funding

Medical writing support and publication fees were funded by PharmaMar. The sponsor had no role in the study design, data collection, data analysis, or interpretation of the data. The sponsor did not influence the content of the manuscript or the decision to submit it for publication Institutional Review Board Statement: The study was conducted in accordance with the Declaration of Helsinki, and approved by the the Ethics Committee for Research with Medicinal Products of the Basque Country (CEIm-Euskadi, EOM2025011) in 13 March 2025.

## Declaration of competing interest

The authors declare that they have no known competing financial interests or personal relationships that could have appeared to influence the work reported in this paper.
